# Luteolin from *Gastrodia elata* Ameliorates Solar Dermatitis via Inhibition of the MAPK/JUN Signalling Pathway

**DOI:** 10.3390/ph19071042

**Published:** 2026-07-03

**Authors:** Yewei Huang, Saizhen Guo, Shundan Li, Ming Zhang, Lijun Cheng, Huan Zhang, Haoyang Li, Yongbin Mai, Jun Sheng, Ruixue Wang, Yongkai Xi

**Affiliations:** 1 College of Science, Yunnan Agricultural University, Kunming 650201, China; lichuangyewei100@163.com (Y.H.); 15608808921@163.com (S.G.); 18445683960@163.com (M.Z.); 18388334615@163.com (H.Z.); wcqfez@163.com (H.L.); maiyongbin2025@163.com (Y.M.); 2 College of Food Science and Technology, Yunnan Agricultural University, Kunming 650201, China; 3 Key Laboratory of Puer Tea Science, Ministry of Education, Yunnan Agricultural University, Kunming 650201, China; shengj@ynau.edu.cn; 4 Yunnan Key Laboratory of Gastrodia and Fungi Symbiotic Biology, Zhaotong University, Zhaotong 657000, China; chenglijun224@ztu.edu.cn; 5 College of Resources and Environment, Yunnan Agricultural University, Kunming 650201, China

**Keywords:** *Gastrodia elata*, solar dermatitis, luteolin, network pharmacology, metabolomics

## Abstract

**Background**: Solar dermatitis (SD) is an inflammatory skin disease caused by excessive exposure to ultraviolet (UV) radiation. *Gastrodia elata* (GE) is a medicinal and edible plant with broad pharmacological activities; however, the polarity distribution of its active components and their specific mechanisms of action in SD remain incompletely understood. **Methods**: Different polarity fractions of GE-petroleum ether extract (PEE), ethyl acetate extract (EAE), and *n*-butanol extract (NBAE) were prepared and evaluated in a UVB-induced SD mouse model. We integrated metabolomics, network pharmacology, molecular docking, molecular dynamics simulations, and molecular biology techniques to identify key active ingredients and regulatory mechanisms. **Results**: The EAE group significantly ameliorated epidermal thickening and collagen damage in SD mice. Mechanistically, EAE and its active component luteolin (LUT) likely suppressed abnormal activation of the JUN pathway (binding energy: −9.1 kcal/mol), leading to downregulation of the pro-inflammatory cytokines tumour necrosis factor-alpha and inerleukin-1 beta. **Conclusions**: The EAE fraction alleviates SD through multi-component, multitarget synergistic effects, with LUT as a core bioactive component that inhibits the JUN pathway to mitigate skin inflammation and oxidative damage. EAE also accelerated SD recovery by modulating critical metabolic pathways, including arginine biosynthesis and terpenoid backbone biosynthesis. These findings identify EAE and LUT as promising candidate therapeutics for SD.

## 1. Introduction

Solar dermatitis (SD) is a common form of photodamage resulting from excessive exposure to ultraviolet (UV) radiation, particularly within the UVB (280–320 nm) spectrum [1]. UVB radiation can directly damage epidermal keratinocyte DNA and induce overproduction of reactive oxygen species, triggering a cascade of pathological processes including oxidative stress, release of inflammatory cytokines, and immune dysregulation. These events ultimately lead to acute inflammatory skin reactions characterised by erythema, oedema, burning, and pain [2]. With increasing global UV radiation intensity and the prevalence of outdoor activities, the incidence of SD is rising annually. This condition not only impairs patients’ quality of life but also, upon recurrent episodes, may elevate the risk of skin carcinogenesis [3]. Current clinical management of SD primarily relies on topical corticosteroids, non-steroidal anti-inflammatory drugs, and physical sunscreens. Although these interventions can alleviate symptoms to some extent, their long-term use is associated with adverse effects such as skin atrophy, telangiectasia, and steroid-dependent dermatitis [4,5]. Consequently, the search for new therapeutic agents that are safe and effective and have minimal side effects represents a crucial direction in dermatological research. Natural products, especially those with multi-target modulatory properties, are of particular interest in this pursuit.

Traditional Chinese Medicine (TCM) offers extensive experience in preventing and treating skin disorders. Its holistic approach, emphasising syndrome differentiation and systemic regulation, provides unique advantages in alleviating inflammation and repairing the skin barrier [6,7]. Numerous herbal medicines and their bioactive constituents have been shown to mitigate UV-induced skin damage through anti-inflammatory, antioxidant, and immunomodulatory activities [8,9].

*Gastrodia elata* (GE), a precious Chinese medicinal plant with dual food and medicinal properties, was first documented in the ancient herbal compendium *Shen Nong’s Classic of Materia Medica*. It is renowned for its efficacy in calming the liver, suppressing hyperactivity, and dispelling wind to unblock meridians [10]. Modern pharmacological studies reveal that GE is rich in bioactive compounds including gastrodin, p-hydroxybenzyl alcohol, and polysaccharides, and various phenolic compounds, which confer anti-inflammatory, antioxidant, and neuroprotective effects [11,12].

Recent studies have highlighted the potential of *Gastrodia elata* extract (GEE) and its constituents in skin protection. For example, gastrodin has been shown to suppress the expression of inflammatory mediators such as matrix metalloproteinase-1 (MMP-1) and tumour necrosis factor-α (TNF-α) in keratinocytes following UVB irradiation, while concurrently upregulating the expression of skin barrier-related proteins including aquaporin 3 (AQP3) and filaggrin (FLG) [13,14]. GE polysaccharide hydrogel can effectively relieve UVB skin damage by acting as an antioxidant, reducing inflammation, and regulating mitochondrial apoptosis pathways [15]. Rhein−Gastrodin Ester-Loaded nanosystems can promote epidermal regeneration and collagen deposition, effectively helping wounds from bacterial infections heal [16]. In vivo studies also confirm that gastrodin derived from Armillaria mellea can ameliorate morphological abnormalities and reduce the degradation of collagen and elastic fibres in UVB-damaged mouse skin [17]. Furthermore, GEE has been reported to attenuate UVA-induced photoaging in human dermal fibroblasts by modulating antioxidant activity and the expression of pro-collagen I, MMP-1, and elastase-1 [18]. Additionally, GEE and its compound formulations can promote HaCaT cell migration and inhibit the excessive secretion of IL-6 and TNF-α induced by UVB [19]. Nevertheless, systematic research comparing the activity and elucidating the mechanisms of different polar fractions derived from GEE in the context of skin photodamage, particularly for SD, remains limited. Therefore, this study aimed to prepare various polar fractions of GEE, evaluate their anti-SD activity, and identify the most effective fraction, thereby providing a theoretical foundation for the development of topical GE-based medications.

The inherent chemical complexity of traditional Chinese medicines poses challenges for systematically unravelling their pharmacological mechanisms using conventional methods. In recent years, with advancements in computational technology, research strategies for investigating drug–disease interactions have evolved from focusing on single targets and isolated compounds towards a more holistic analysis of a drug’s regulatory effects on biomolecular networks [20]. Network pharmacology, an interdisciplinary field integrating systems biology and network informatics, has demonstrated significant advantages in the discovery of new drugs and exploration of their mechanisms, particularly suited for the multi-component, multi-target nature of herbal medicines [21].

The workflow of this study is summarised as follows. First, GEE was fractionated using solvent partitioning to obtain extracts of varying polarity (petroleum ether extract, PEE; ethyl acetate extract, EAE; *n*-butanol extract, NBAE). The anti-SD activity of these fractions was compared through in vivo efficacy experiments to screen the most active fraction. Subsequently, an integrated approach employing untargeted metabolomics, network pharmacology, molecular docking, and molecular dynamics simulations was used to predict and identify the key bioactive constituents, core therapeutic targets, and signalling pathways associated with the active fraction. Finally, the efficacy of the identified core active compound was validated both in vitro and in vivo using an SD mouse model and an HaCaT cell damage model. The underlying molecular mechanism, centred on the predicted target, was further investigated. This study clarifies the material basis and mechanism of action of GE against SD, providing a scientific basis for developing novel topical anti-photodamage agents based on GE’s active constituents.

## 2. Results and Discussion

### 2.1. EAE from GEE Exhibits Remarkable Therapeutic Effects on SD

**Figure 1:**
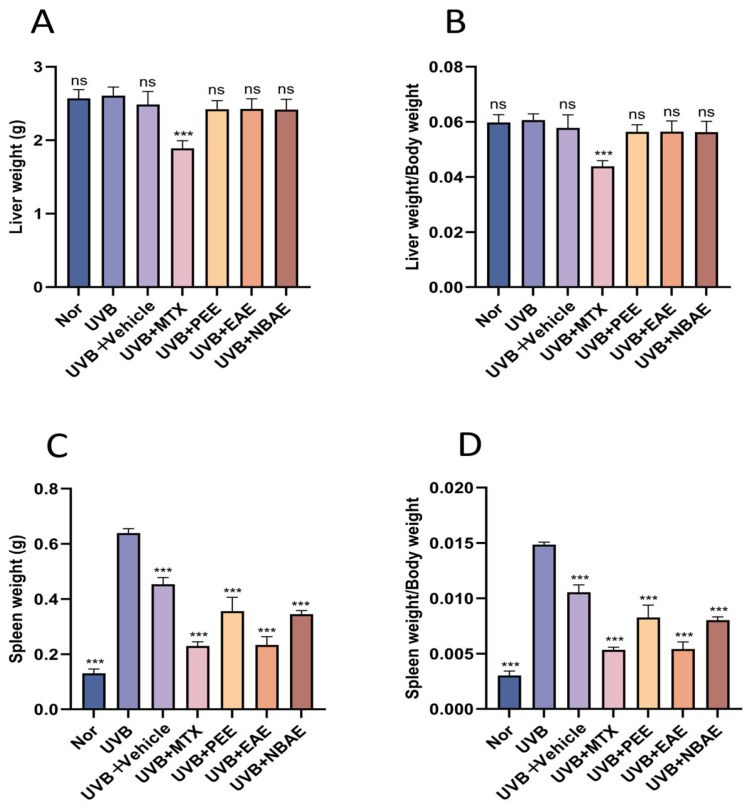
Therapeutic effects of GEE polarity fractions on SD pathology. (**A**,**B**) Liver weight and liver index. (**C**,**D**) Spleen weight and spleen index. Data are expressed as mean ± SEM (*n* = 8) (*** *p* < 0.001; ns: not significant (vs. model group)).

In this study, the therapeutic efficacy of different polarity fractions from GEE on SD in KM mice was evaluated following 12 days of topical treatment. SD mice exhibited typical skin inflammatory responses as previously described, including increased back skin thickness, wrinkle formation, and dryness [22]. The results showed that, compared to PEE and NBAE, EAE and the positive control MTX group significantly improved dermatitis symptoms (Supplementary Figure S1). Compared to the UVB group, all GEE polarity fractions showed favourable safety profiles in terms of liver weight and liver index (Figure 1A,B). The spleen weight and spleen index of mice in the EAE treatment group were significantly reduced compared to the UVB group, and EAE demonstrated superior improvement compared to PEE and NBAE (Figure 1C,D).

**Figure 2:**
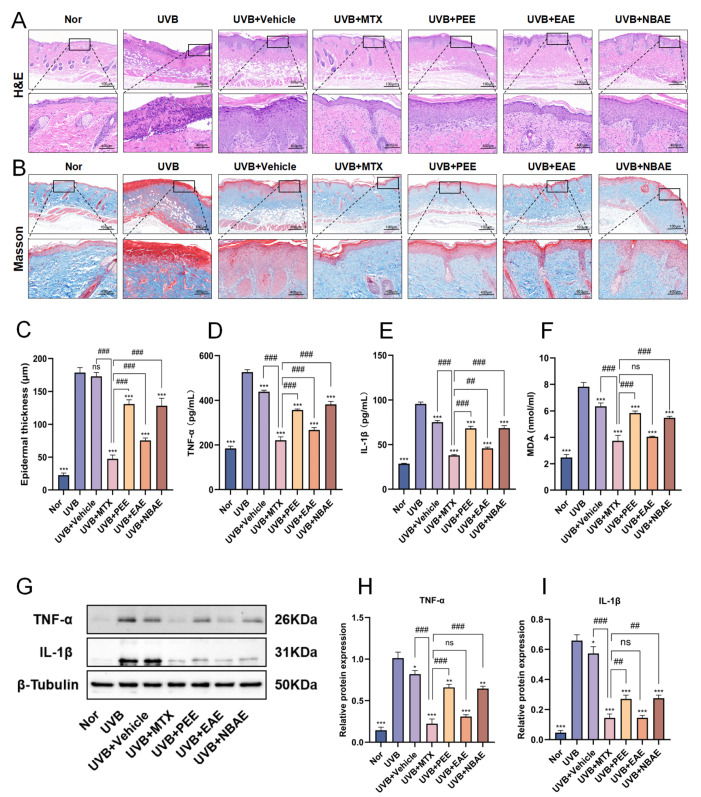
The EAE fraction ameliorates cutaneous histopathology and suppresses inflammatory mediators in SD mice. (**A**,**B**) Representative H&E and Masson’s trichrome staining of dorsal skin sections after 12 days of treatment. (**C**) Epidermal thickness quantification post-treatment. (**D**–**F**) Levels of TNF-α, IL-1β, and MDA in skin tissues quantified by ELISA. (**G**) Western blot analysis of TNF-α and IL-1β signalling proteins in UVB-irradiated dorsal tissues. (**H**,**I**) Densitometric quantification of TNF-α and IL-1β protein expression. Data are expressed as mean ± SEM (*n* = 3) (* *p* < 0.05, ** *p* < 0.01, *** *p* < 0.001 compared with the model group; ^##^ *p* < 0.01, ^###^ *p* < 0.001 compared with the UVB + MTX group; ns, no significant difference).

Significant epidermal hyperplasia and inflammatory cell infiltration are hallmarks of SD mice [23]. To investigate the specific improvements in EAE and other polarity fractions on different skin layers after UVB exposure, H&E staining was performed on mouse skin. The results revealed that the UVB-induced group exhibited typical pathological features of SD, including epidermal thickening, hyperkeratosis, and dermal necrosis. After intervention with different GEE polarity fractions, EAE treatment showed reduced inflammatory cell infiltration in the epidermis and a more regular arrangement of keratinocytes (Figure 2A). Masson’s staining further indicated that EAE treatment restored the UVB-induced reduction in and disorganisation of collagen fibres (Figure 2B). ELISA assays demonstrated that EAE was significantly more effective than PEE and NBAE in reducing the levels of pro-inflammatory factors TNF-α and IL-1β and the oxidative stress marker MDA (Figure 2D–F). Western blot results further confirmed its inhibitory effect on TNF-α and IL-1β (Figure 2G–I).

### 2.2. JUN Is a Crucial Target for the Therapeutic Effects of EAE on SD

To explore the reason for the superior therapeutic effect of EAE on SD compared to other polarity fractions, we systematically analysed the different polarity fractions of GEE using non-targeted metabolomics. PCA analysis of PEE, EAE, and NBAE is shown in Supplementary Figure S2. Representative total ion chromatograms (TIC) in negative and positive ion modes are provided in Supplementary Figure S3 and Supplementary Figure S4, respectively. In total, 448, 441, and 307 non-volatile secondary metabolites were detected, respectively, and relative quantification and classification were performed for each polarity fraction, resulting in 11 major categories. The results indicated that phenylpropanoids and polyketides constituted a large proportion in EAE, suggesting they might be the primary components responsible for treating SD (Supplementary Figure S5).

**Figure 3:**
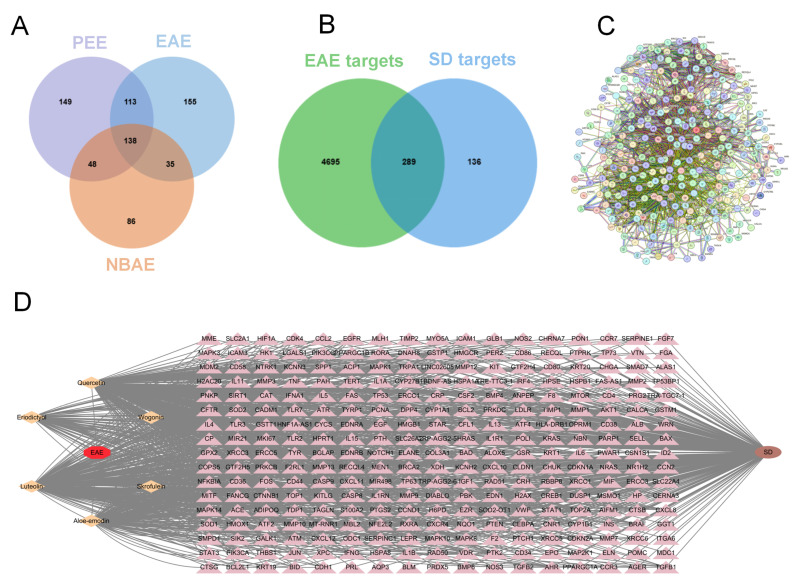
Potential overlapping targets of the EAE group in treating SD. (**A**) Venn diagram of three different fractions. (**B**) Bioactive components of the EAE group Venn diagram of targets related to SD. (**C**) PPI network of the 289 targets for EAE group in treating SD. (**D**) Compound–target–disease network diagram, where diamonds represent the major bioactive components of the EAE group, and triangles indicate the common targets between the EAE group and SD.

Venn analysis revealed common and unique components among the three fractions. From the 155 unique components identified exclusively in EAE (Figure 3A), we applied a two-step filtering strategy to prioritise candidate bioactive compounds. First, all unique components were subjected to ADMET screening using the TCMSP database with dual thresholds of oral bioavailability (OB) ≥ 30% and drug-likeness (DL) ≥ 0.18, which are widely accepted criteria for identifying drug-like compounds with favourable pharmacokinetic properties [24]. This initial screening reduced the candidate pool to six compounds: aloe-emodin, eriodictyol, luteolin, quercetin, skrofulein, and wogonin (Table 1). These six compounds, primarily belonging to phenylpropanoids and polyketides, were selected for subsequent target prediction and molecular docking analyses due to their optimal pharmacokinetic profiles among all EAE-exclusive components. Integrated analysis using BATMAN-TCM, HERB, CTD, TCMSP, CHEMBL, and SwissTarget Prediction databases yielded 4984 potential targets. SD-related targets were obtained from the GeneCards, OMIM, and DrugBank databases, totalling 425. Intersection with SD-related targets identified 289 key targets (Figure 3B). The STRING database constructed a protein–protein interaction (PPI) network containing these 289 potential therapeutic targets, comprising 289 nodes and 2510 edges (Figure 3C). A “compound–target” interaction network was constructed (Figure 3D).

**Figure 4:**
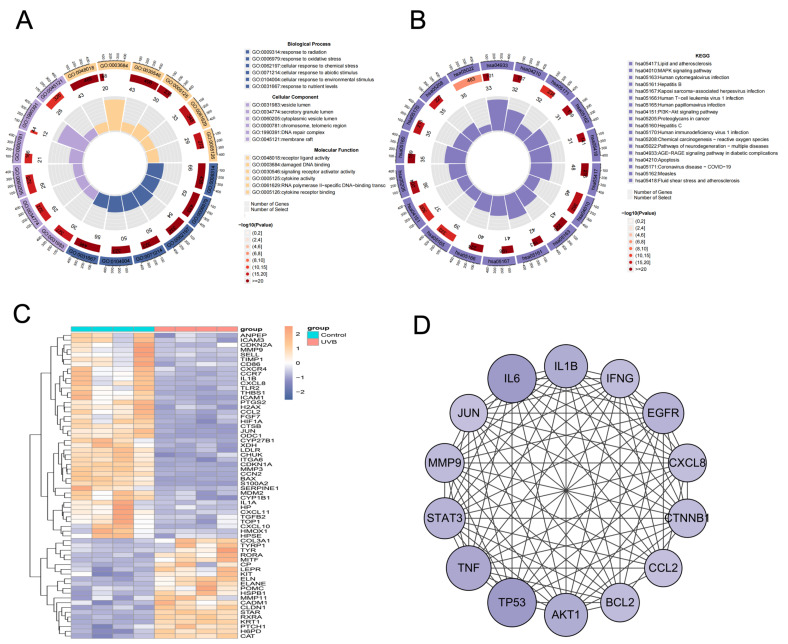
JUN as a key target gene for the EAE group in treating SD. (**A**) GO enrichment analysis of targets for the EAE group in treating SD. (**B**) KEGG pathway enrichment analysis of targets for the EAE group in treating SD. (**C**) Heatmap of the intersection between the GSE54413 dataset and the 289 targets for the EAE group in treating SD. (**D**) Visualisation of core targets for the EAE group in treating SD.

GO enrichment analysis revealed the top six significantly enriched biological processes (BP), including response to radiation (GO:0009314) and response to oxidative stress (GO:0006979); cellular components were mainly enriched in the vesicle lumen (GO:0031983), and molecular functions (MFs) were most significant in receptor ligand activity (GO:0048018) (Figure 4A). KEGG pathway analysis further identified 18 key pathways, including lipid and atherosclerosis (hsa05417) and the MAPK signalling pathway (hsa04010) (Figure 4B). Analysis integrating the GSE54413 dataset revealed 2489 differentially expressed genes (DEGs) (|log_2_ FC| > 1, adjusted *p*-value < 0.05) in SD patient lesion tissues, including 1139 upregulated and 1350 downregulated genes (Supplementary Figure S6). The intersection of upregulated genes with the 289 targets yielded 66 key genes (Figure 4D). Further cross-validation with the top 14 core targets from the PPI network ultimately identified JUN as the key hub gene for EAE treatment of SD (Figure 4D).

### 2.3. LUT Is Likely the Major Functional Component in EAE for Treating SD

**Figure 5:**
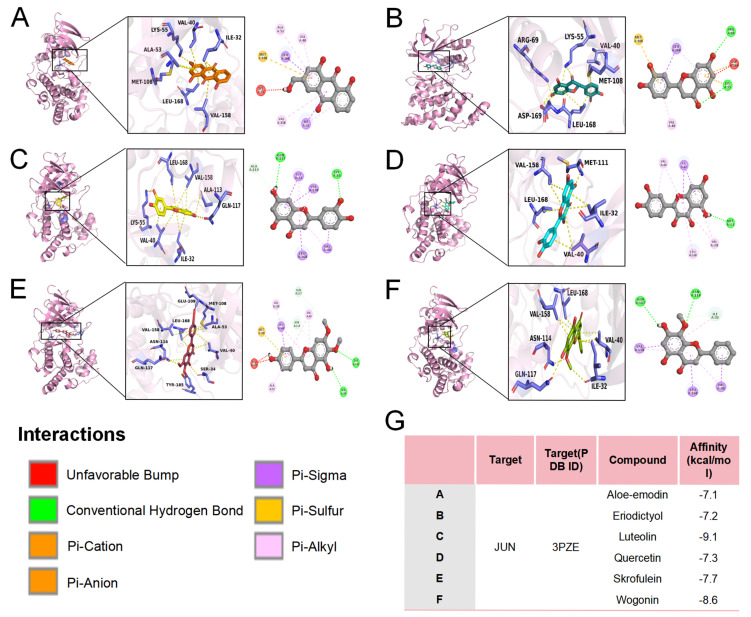
Luteolin in the EAE group exhibits favourable binding energy with JUN protein. (**A**–**F**) Three-dimensional schematic diagrams of JUN binding with aloe-emodin, eriodictyol, luteolin, quercetin, skrofulein, and wogonin, respectively. (**G**) Docking scores of JUN protein with the six bioactive components.

This study systematically evaluated the interaction mechanism between EAE active components and the hub gene JUN using computational simulation methods. Molecular docking analysis of the JUN protein crystal structure (PDB ID: 3PZE) with the six active compounds (quercetin, wogonin, eriodictyol, luteolin, skrofulein, and aloe-emodin) was performed using Autodock-Vina-1.5.6 software [25]. The results showed that luteolin had the optimal binding affinity with JUN (Figure 5G). By extracting ion chromatograms and performing primary mass spectrometry analysis (see Supplementary Figure S7), the presence of luteolin in EAE was further confirmed.

**Figure 6:**
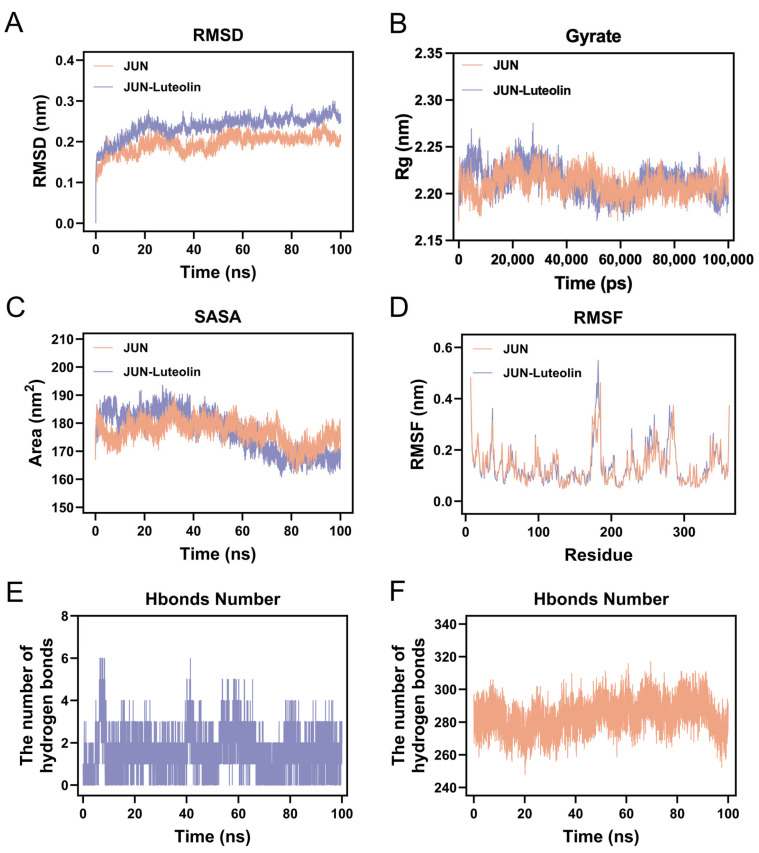
LUT exhibits strong binding stability with JUN protein. (**A**) RMSD of JUN protein and the complex during MDS. (**B**) Radius of gyration variation in JUN protein and the complex during MDS. (**C**) Solvent-accessible surface area (SASA) of JUN protein and the complex during MDS. (**D**) Root-mean-square fluctuation (RMSF) of JUN protein and the complex during MDS, showing a larger fluctuation range on the surface. (**E**) Number of hydrogen bonds (HBNUM) between JUN protein and the complex during MDS. (**F**) Number of hydrogen bonds (HBNUM) between JUN protein and water molecules. (The term “protein” refers to JUN protein, and “complex” refers to the JUN protein and luteolin ligand system).

To verify the dynamic stability of the luteolin–JUN protein complex, we performed molecular dynamics simulations (MDS). Analysis of kinetic parameters showed that the root-mean-square deviation (RMSD) of the system stabilised after 15 ns (Figure 6A), indicating the complex structure reached equilibrium. Furthermore, the fluctuation trends of the six simulation datasets were consistent with RMSD changes. The JUN system exhibited good structural stability in both the RMSD and radius of gyration analyses (Figure 6B). During the 0 to 100 ns simulation, solvent accessible surface area (SASA) analysis showed that the surface exposure index of the JUN protein remained at a low level (Figure 6C), further confirming the stability of its overall structure. Root-mean-square fluctuation (RMSF) analysis was used to examine the dynamic characteristics of local conformations within the system (Figure 6D), revealing low conformational fluctuations in key interaction regions of the complex. Notably, hydrogen bonds, as the primary force maintaining protein–ligand complex stability, exhibited dynamic changes during the simulation. Specifically, the number of hydrogen bonds between JUN and luteolin ranged from 1 to 6 (Figure 6E,F), providing direct evidence for complex stability at the molecular interaction level.

### 2.4. Functional Validation of LUT for SD Treatment at the Cellular Level

**Figure 7:**
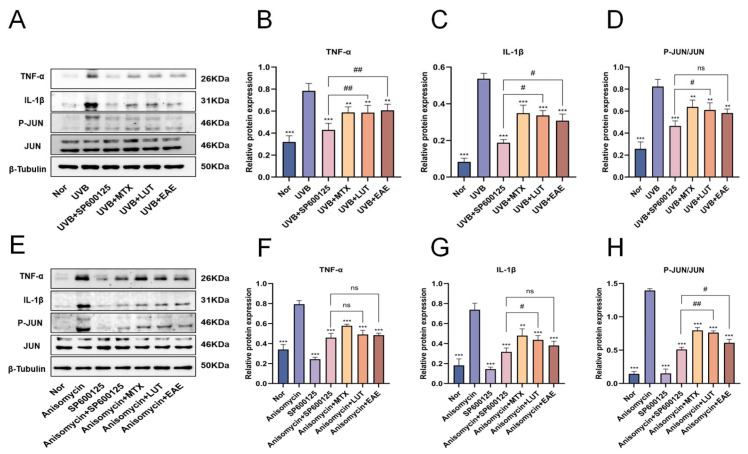
Effects of EAE and LUT on the JUN signalling pathway in UVB-induced HaCaT cells (**A**–**D**). Western blot images (**A**) and relative expression levels of TNF-α (**B**), IL-1β (**C**), and P-JUN/JUN (**D**) in cells. Effects of EAE and LUT on the JUN signalling pathway in HaCaT cells induced by JUN activator (**E**–**H**). Western blot images (**E**) and relative expression levels of TNF-α (**F**), IL-1β (**G**), and P-JUN/JUN (**H**) in cells. Data are expressed as mean ± SEM (*n* = 3) (** *p* < 0.01, *** *p* < 0.001 compared with the UVB and Anisomycin group; # *p* < 0.05, ## *p* < 0.01 compared with the UVB + SP600125 and Anisomycin + SP600125 group; ns, no significant difference).

KEGG pathway analysis indicated the MAPK signalling pathway was the primary enriched pathway. We investigated the inhibitory effect of LUT on P-JUN using UVB irradiation and a JUN-specific inhibitor. The MTT assay results showed that both EAE and LUT inhibited the proliferation of HaCaT cells in a concentration-dependent manner (Supplementary Figures S8 and S9). The results demonstrated that EAE and LUT simultaneously downregulated the UVB-induced overexpression of pro-inflammatory factors TNF-α and IL-1β. Notably, treatment with EAE and LUT exhibited an inhibitory effect similar to that of the JUN-specific inhibitor, significantly reducing JUN phosphorylation levels (Figure 7A–D). To further verify this mechanism, intervention experiments were performed using the JUN activator anisomycin and the inhibitor SP600125. The results showed that both EAE and LUT effectively antagonised the activation by anisomycin, and their inhibitory effects were superior to the positive control MTX group (Figure 7E–H). These results confirm at the molecular level that EAE and LUT alleviate SD by specifically inhibiting the activation of the JUN signalling pathway.

### 2.5. Functional Validation of LUT for SD Treatment at the Animal Level

**Figure 8:**
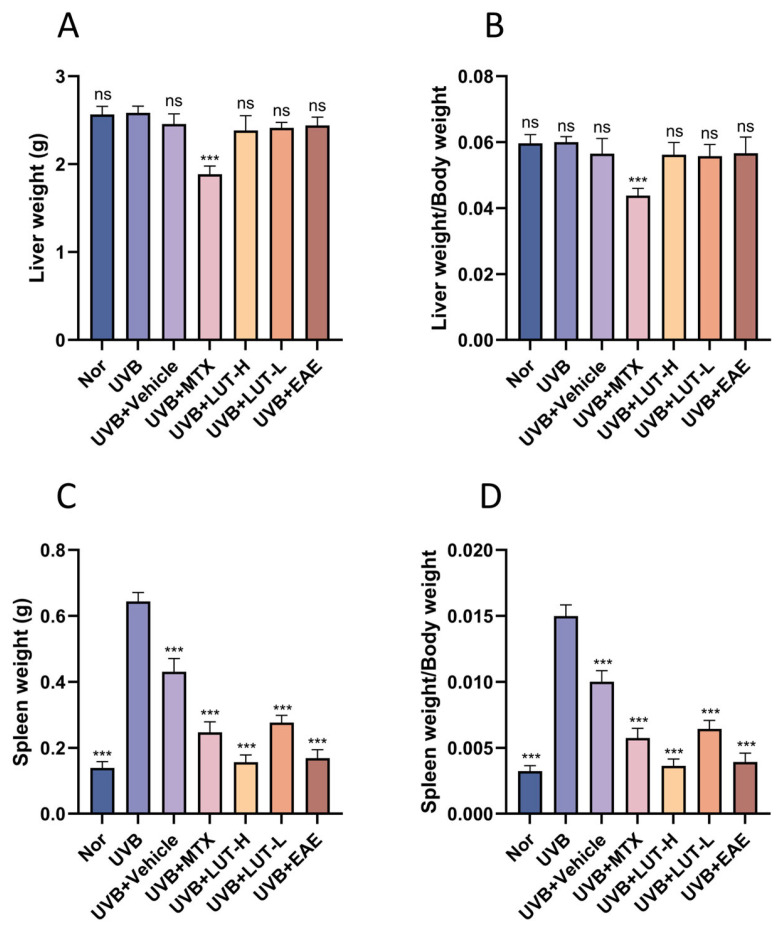
LUT ameliorated SD skin lesions. (**A**,**B**) Liver weight and liver index of mice. (**C**,**D**) Spleen weight and spleen index of mice. Data are expressed as mean ± SEM (*n* = 8). *** *p* < 0.001 and ns indicates no significance, compared with the UVB group.

To evaluate the therapeutic effect of LUT on SD, we recorded changes in the dorsal skin of mice 3 days after UVB irradiation. KM mice exhibited typical skin inflammatory responses as previously described, including increased back thickness, wrinkle formation, and dryness. After 12 days of topical treatment, the UVB + LUT, UVB + MTX, and UVB + EAE groups all showed significant improvement in dermatitis symptoms (Supplementary Figure S10). Organ index analysis revealed that compared to the UVB-induced group, the spleen index was significantly reduced in the UVB + LUT and UVB + EAE groups, with no significant liver toxicity observed. In contrast, the UVB + MTX group exhibited significant liver damage. The UVB + Vehicle group also showed a slight improvement in dermatitis symptoms (Figure 8A–D). These results indicate that LUT not only effectively alleviates UVB-induced skin inflammation but also demonstrates a superior safety profile compared to the conventional therapeutic drug MTX.

**Figure 9:**
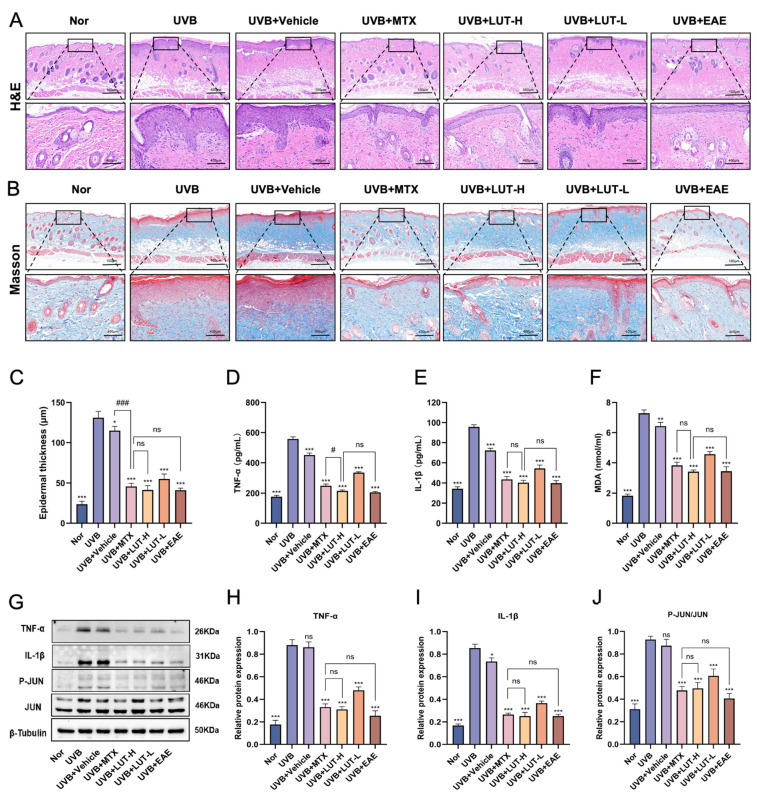
LUT ameliorates skin pathological damage and reduces the expression of inflammatory cytokines in SD mice. H&E and Masson staining of mouse skin from each group after 12 days of SD exposure (**A**,**B**). (**C**) Thickness of the epidermal layer of mouse skin after 12 days of SD exposure. Levels of TNF-α (**D**), IL-1β (**E**), and MDA (**F**) in mouse skin were detected using ELISA kits. Western blot images (**G**) and relative expression levels (**H**–**J**) of TNF-α, IL-1β, P-JUN, and JUN signalling pathway proteins in the dorsal tissue of UVB-induced SD mice treated with various drugs. Data are expressed as mean ± SEM (*n* = 3) (* *p* < 0.05, ** *p* < 0.01, *** *p* < 0.001 compared with the model group; # *p* < 0.05, ### *p* < 0.001 compared with the UVB + MTX and UVB + LUT-H groups; ns, no significant difference).

The H&E staining results showed that LUT intervention significantly improved epidermal hyperplasia and inflammatory cell infiltration and restored normal epidermal structural arrangement (Figure 9A,C). Masson’s trichrome staining further revealed that compared to the normal group, the number of blue collagen fibres in the dermis was markedly reduced and disorganised in the UVB-induced and UVB + Vehicle groups. In contrast, the LUT-treated group exhibited an increased number of blue-stained fibres and more organised collagen arrangement in the dermis, indicating that LUT can prevent UVB-induced destruction of collagen fibres in KM mouse skin (Figure 9B). At the molecular mechanism level, ELISA assays showed that LUT significantly reduced the levels of pro-inflammatory factors TNF-α and IL-1β and the oxidative stress marker MDA in skin tissues. Notably, there was no significant difference in the therapeutic effect on inflammatory factor expression among the UVB + LUT-H, UVB + MTX, and UVB + EAE groups (Figure 9D–F). Western blot analysis revealed that LUT exerts its therapeutic effect by regulating the MAPK signalling pathway, significantly inhibiting JUN protein phosphorylation levels while downregulating the expression of TNF-α and IL-1β (Figure 9G–J). These results systematically elucidate, from histopathology to the molecular level, the mechanism by which LUT improves UVB-induced skin damage by inhibiting MAPK/JUN signalling pathway activation, thereby reducing oxidative stress and inflammatory responses.

### 2.6. LUT Ameliorates SD Changes in Skin Metabolites

**Figure 10:**
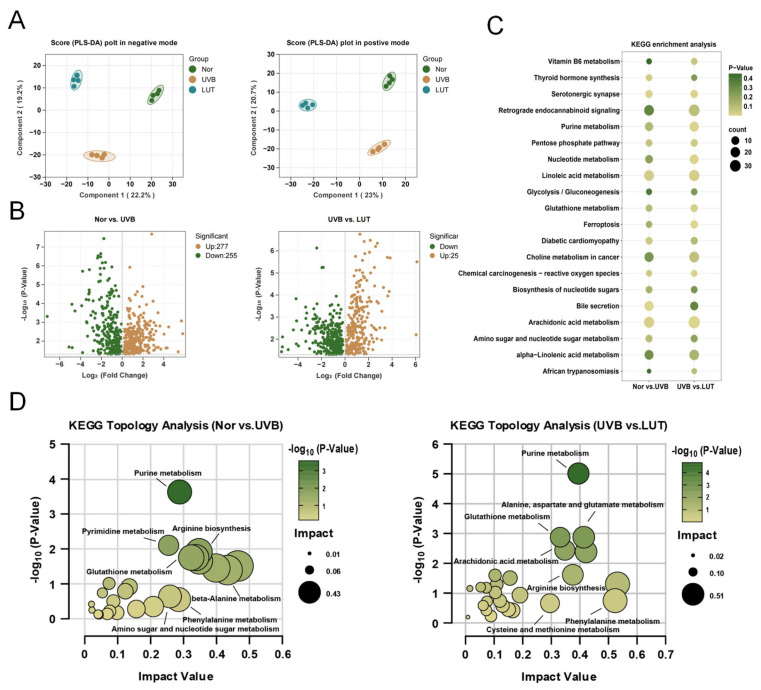
LUT ameliorates UVB-induced changes in the skin metabolome of mice. (**A**) PLS-DA score plot of metabolomics features among groups. (**B**) Volcano plot of differentially expressed metabolites among groups, with red dots indicating upregulated metabolites and green dots indicating downregulated metabolites. (**C**) KEGG pathway enrichment analysis of differentially expressed metabolites among groups. (**D**) KEGG topological analysis of differentially expressed metabolites. Circles represent different KEGG pathways, with the size of the circle indicating the number of differentially expressed metabolites in the pathway and the colour indicating the *p*-value (i.e., the significance level in enrichment analysis). Metabolomics feature analysis (*n* = 4).

Non-targeted metabolomics with PLS-DA analysis was performed on KM mouse skin tissues to investigate the impact of LUT on SD metabolic alterations. Clear separation of metabolite profiles among the Normal, UVB, and LUT groups was observed in both positive and negative ESI modes (Figure 10A). Between the Normal and UVB groups, 2069 differentially expressed metabolites (DEMs) were identified (*p* < 0.05, VIP > 1; 277 up-, 255 downregulated). Similarly, 2069 DEMs were found between the UVB and LUT groups (254 up-, 281 downregulated) (Figure 10B).

KEGG enrichment analysis revealed that UVB irradiation significantly activated pathways associated with oxidative stress and inflammation (e.g., chemical carcinogenesis—reactive oxygen species, arachidonic acid metabolism, ferroptosis) while suppressing protective pathways like glutathione metabolism. LUT treatment effectively reversed these changes, upregulating antioxidant pathways (glutathione metabolism, pentose phosphate pathway) and downregulating inflammatory pathways (arachidonic acid metabolism) (Figure 10C).

Topological analysis further identified 16 significantly altered pathways between the Normal and UVB groups (*p* < 0.05, impact > 0.1), including purine metabolism, glutathione metabolism, and arachidonic acid metabolism. In the UVB vs. LUT comparison, 22 pathways showed significant reversal, including not only the aforementioned pathways but also others such as one-carbon pool by folate and terpenoid backbone biosynthesis, which were not significantly altered by UVB alone (Figure 10D). These results suggest that LUT ameliorates UVB-induced photodamage by restoring metabolic homeostasis, modulating redox balance, and suppressing inflammatory responses through multiple metabolic pathways.

## 3. Discussion

SD is an inflammatory skin disease induced by UVB radiation, which may progress to skin cancer upon long-term and repeated exposure [26]. Currently, there is a lack of ideal therapeutic agents for SD. Traditional Chinese Medicine (TCM) offers a unique perspective for the prevention and treatment of SD. Its therapeutic approach extends beyond merely alleviating superficial symptoms, emphasising the regulation of internal Yin–Yang balance and promoting the smooth flow of Qi and blood, reflecting a holistic treatment philosophy [27]. In recent years, there has been growing interest in screening active components from Chinese herbal medicines for disease treatment. TCM has made significant progress in the prevention and treatment of SD, demonstrating anti-inflammatory, antioxidant, anti-photoaging, and skin barrier repair effects, as well as effectively reducing UV-induced skin damage [28].

GE exhibits diverse pharmacological activities, including anti-angiogenic, anti-inflammatory, anti-cancer, anti-diabetic, hypotensive, and neuroprotective effects [29]. Studies suggest these activities are closely related to its rich content of phenolic and flavonoid compounds [30]. In recent years, GE and its monomeric compounds have been extensively investigated as anti-inflammatory agents [31]; however, the polar distribution characteristics of its active components for treating SD and their specific mechanisms of action have not been fully elucidated.

In this study, solvent gradient extraction was employed to fractionate the total GEE, yielding PEE, EAE and NBAE fractions to evaluate which polar segment primarily contains the anti-SD active components. The results indicated that EAE exhibited the strongest therapeutic activity. Secondary metabolite analysis of EAE identified six potential active components and 289 SD-related therapeutic targets, suggesting that quercetin, wogonin, eriodictyol, luteolin, skrofulein, and aloe-emodin may be the main active components in EAE for treating SD. The literature supports the significant anti-inflammatory effects of these components: wogonin can exert therapeutic effects in UVB-induced SD models by inhibiting the expression of TNF-α, IL-6, EGFR, MMP9, and IL-17 [32]. Quercetin exhibits anti-inflammatory effects in MC903-induced atopic dermatitis models by suppressing the expression of CCL17, CCL22, IL-4, IL-6, IFN-γ, and TNF-α [33], and can improve inflammation, oxidative stress, and wound healing impairment in human keratinocytes via the MAPK and NF-κB pathways [34]; it may also influence the STAT1 signalling pathway by modulating IFN-γ and TNF-α in HaCaT cells [35]. Luteolin can exert intestinal anti-inflammatory effects by inhibiting the JAK/STAT pathway [36], and improve renal inflammation in diabetic nephropathy by suppressing the NF-κB and TGF-β1/Smad3 pathways and modulating the STAT3 pathway [37]. Eriodictyol can alleviate inflammation in IL-1β-induced human osteoarthritis chondrocytes by inhibiting lipid raft formation and the PI3K/AKT/NF-κB signalling pathway [38]; studies have reported that aloe-emodin-loaded chitin nanogel shows anti-psoriatic activity in a mouse tail model [39]. This evidence collectively indicates that the main active components in the EAE fraction possess significant anti-inflammatory potential.

GO functional analysis revealed that these targets are primarily associated with biological processes such as response to radiation, response to oxidative stress, the vesicle lumen, the secretory granule lumen, receptor ligand activity, and damaged DNA binding. KEGG pathway analysis suggested that the active compounds in EAE may treat SD through multiple pathways, including lipid metabolism and atherosclerosis, the MAPK signalling pathway, human cytomegalovirus infection, hepatitis B, the PI3K–Akt signalling pathway, and the AGE–RAGE signalling pathway (Figure 4A,B). PPI network topology analysis further indicated that TP53, TNF, STAT3, MMP9, JUN, IL1B, IFNG, and EGFR, among others, may be key targets for these active components in treating SD (Figure 4D).

Integrating microarray data analysis, we identified 2489 DEGs in SD patient lesional tissues from the GEO dataset GSE54413, including 1139 upregulated and 1350 downregulated genes. Intersecting these DEGs with the 289 targets from the PPI network yielded 66 key targets (Figure 4D). Further intersection with the core PPI targets ultimately identified JUN as the key hub gene. Molecular docking analysis demonstrated that LUT had the highest binding affinity for JUN (Figure 5). Molecular dynamics simulation results further confirmed the stable binding conformation of the LUT–JUN complex (Figure 6), providing a basis for understanding protein conformational changes and functional relationships induced by ligand binding. However, computational predictions alone cannot establish biological efficacy; therefore, we sought to validate the JUN-targeting hypothesis through sequential in vitro and in vivo experiments.

The convergence of computational predictions with experimental validations constitutes a central strength of this study. Network pharmacology screening initially reduced 155 EAE-exclusive metabolites to six candidates with favourable ADMET properties, and subsequent PPI topology analysis cross-referenced with the GSE54413 dataset identified JUN as the highest-priority hub target. Molecular docking and 100 ns molecular dynamics simulations provided biophysical evidence that luteolin stably engages the JUN protein (PDB: 3PZE) with optimal binding energy (−9.1 kcal/mol) and sustained hydrogen-bond interactions. These in silico findings were functionally tested in UVB-irradiated HaCaT keratinocytes: both EAE and luteolin significantly suppressed JUN phosphorylation and downstream TNF-α and IL-1β expression, effectively phenocopying the JUN-specific inhibitor SP600125. Importantly, in anisomycin-induced JUN hyperactivation models, luteolin antagonised pathway overstimulation, confirming target-specific engagement rather than generalised cytoprotection. Translating these cellular observations to an organismal context, topical luteolin in UVB-induced SD mice recapitulated the molecular phenotype by reducing cutaneous p-JUN, TNF-α, and IL-1β levels while concomitantly ameliorating epidermal hyperplasia and collagen disorganisation. Untargeted metabolomics provided an additional mechanistic layer, demonstrating that luteolin reversed UVB-induced suppression of glutathione metabolism and pentose phosphate pathway activity, thereby coupling the JUN-centric anti-inflammatory effect to the restoration of cellular redox homeostasis. Collectively, these multi-platform data establish a coherent translational framework in which computational target identification and binding validation were sequentially confirmed by cellular pathway pharmacology, animal histopathological efficacy, and systems-level metabolic remodelling.

At the tissue level, these molecular findings were manifested as measurable histopathological improvements. H&E staining revealed that luteolin intervention restored normal epidermal architecture and reduced inflammatory cell infiltration, while Masson’s trichrome staining confirmed the prevention of UVB-induced collagen fibre loss and disorganisation in mouse dermis (Figure 9). ELISA quantification further verified significant reductions in cutaneous TNF-α, IL-1β, and MDA, corroborating the Western blot and cellular data at the biochemical level.

**Figure 11:**
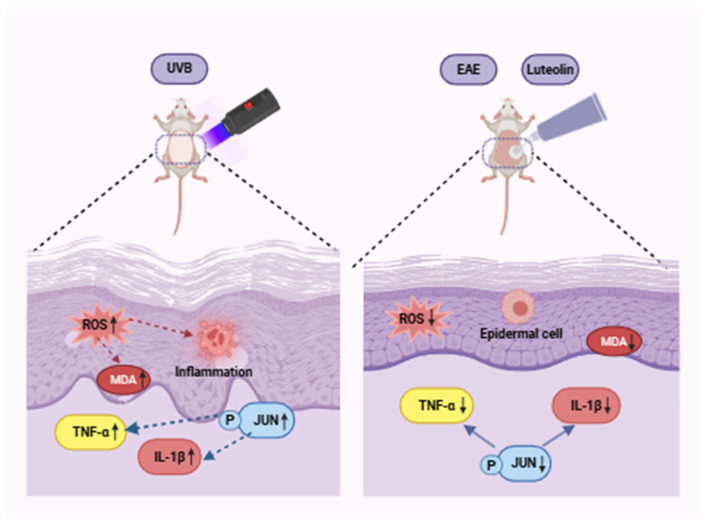
Mechanism of GE in Treating SD.

Aberrant JUN signalling is recognised as a critical driver of UVB-induced cutaneous inflammation. By suppressing JUN phosphorylation, luteolin interrupts this pathogenic cascade, downregulating key inflammatory mediators in both keratinocyte cultures and mouse skin. The metabolomic findings add a further dimension to this mechanism by demonstrating that luteolin concurrently restores glutathione metabolism and pentose phosphate pathway activity (Figure 10), suggesting that anti-inflammatory and antioxidant effects are metabolically coupled. This systems-level insight aligns with the network pharmacology prediction that EAE acts through multi-target, multi-pathway synergy, and consolidates the JUN-centred mechanism illustrated in Figure 11.

There are certain limitations to our survey. First, the main active ingredients of LUT used to treat SD in EAE were identified through methods like network pharmacology and molecular docking, without considering the proportion of these ingredients in EAE. Future lab studies will focus on the ratios of different ingredients in EAE and their effects on SD. Second, while our experiments validated the JUN signalling pathway as a key mechanistic node, the multi-component and multi-target nature of EAE suggests that additional, uncharacterised targets and pathways may contribute to the observed therapeutic effects. Third, the absence of clinical data represents a significant translational gap; the dermal efficacy, optimal dosing, and long-term safety of luteolin and EAE in human solar dermatitis remain to be established through controlled clinical trials. Fourth, the preclinical models employed—HaCaT cells and KM mice with acute UVB exposure—simplify the chronic, relapsing clinical course of human SD and may not fully predict human pharmacokinetics or immunological responses. Finally, the metabolite filtering strategy was constrained by ADMET thresholds (OB ≥ 30%, DL ≥ 0.18), potentially excluding other bioactive constituents with poor predicted oral bioavailability but potent topical activity. Future investigations should address these constraints by incorporating human skin explant models, expanded preclinical pharmacology, and rigorous clinical evaluation.

## 4. Materials and Methods

### 4.1. Reagents and Chemicals

GE was obtained from the Xiaocaoba area, Yiliang County, Zhaotong City, Yunnan Province, China. Petroleum ether (8032-32-4), ethyl acetate (141-78-6), and *n*-butanol (71-36-3) were purchased from Sinopharm Chemical Reagent Co., Ltd. (Shanghai, China). The BCA protein assay kit was sourced from Tianqing Biotech (Beijing, China). PVDF membranes were obtained from Merck Millipore (Burlington, MA, USA). Carbomer 940 was purchased from Solarbio (Beijing, China). Luteolin (LUT) was procured from Innochem (Beijing, China). Methotrexate (MTX) was obtained from Adamas (Shanghai, China). Primary antibodies against JUN (1:1000), phospho-JUN (p-JUN, 1:1000), and TNF-α (1:1000) were obtained from Proteintech (Wuhan, China). The IL-1β antibody (1:1000) was obtained from ABclonal (Wuhan, China). Anisomycin and SP600125 were purchased from MedChemExpress (MCE, Shanghai, China). ELISA kits for detecting tumour necrosis factor-α (TNF-α), interleukin-1β (IL-1β), and malondialdehyde (MDA) were obtained from Duma Biotech Co., Ltd. (Wuhan, China). The 4% paraformaldehyde fixative was obtained from Servicebio (Wuhan, China). Other reagents were of analytical grade.

### 4.2. Sample Collection and Preparation

Fresh GE rhizomes were washed with deionized water, sliced, and dried in a 50 °C oven until a constant weight was achieved. The dried slices were then ground and passed through a No. 60 sieve to obtain a fine powder. A total of 500 g of the powder was extracted with 75% ethanol at a solid-to-liquid ratio of 1:20 (*w*/*v*) by refluxing three times at 85 °C for 2 h each. After each reflux, the mixture was filtered, and the filtrates were combined. The combined extracts were concentrated under reduced pressure to yield the Gastrodia elata ethanol extract (GEE, 88.19 g, yield 17.64%). The GEE was then suspended in distilled water and sequentially partitioned with petroleum ether, ethyl acetate, and *n*-butanol (three times with equal volumes of each solvent). The respective organic layers were combined and concentrated using a rotary evaporator to obtain the petroleum ether extract (PEE, 3.55 g), ethyl acetate extract (EAE, 3.65 g), and *n*-butanol extract (NBAE, 19.43 g). The yields of PEE, EAE, and NBAE were 0.71%, 0.73%, and 3.89%, respectively, calculated based on the initial weight of the raw powder.

### 4.3. Non-Target Metabolomics Analysis by UPLC-Q-TOF/MS

Analysis was performed on an ACQUITY UPLC HSS T3 column (100 mm × 2.1 mm, 1.8 μm), with a column temperature of 45 °C; mobile phase A consisted of water (containing 0.1% formic acid), and mobile phase B was acetonitrile. The gradient was set as follows: 95% mobile phase B for 0–2 min, 95–5% mobile phase B for 2–4 min, 70–30% mobile phase B for 4–8 min, 50–50% mobile phase B for 8–10 min, 20–80% mobile phase B for 10–14 min, and 0–100% mobile phase B for 14–15 min; then mobile phase B was reduced to 95% at 15.1 min and held for a 1 min equilibration period. The injection volume was set to 3 μL, and the flow rate of the mobile phase in the liquid phase was set to 0.35 mL/min.

The parameters were set as follows: spray voltage of 3800 V (positive mode) and −3000 V (negative mode); capillary temperature of 320 °C; aux gas heater temperature of 350 °C; sheath gas flow rate of 35 Arb; aux gas flow rate of 8 Arb; S-lens RF level of 50; mass range (*m*/*z*) of 70–1050 Da; full MS resolution of 70,000; MS/MS resolution of 17,500; NCE/stepped NCE of 10, 20, 40.

The raw data was processed using Progenesis QI software for peak extraction, peak alignment, and compound identification. The identification of small-molecule compounds ranging from 70 to 1050 Da was achieved through the Progenesis QI software online METLIN database and in-house database. The visualisation of the final results was performed using SIMCA software (version 14.1).

### 4.4. Animal Experiments

#### 4.4.1. Animals

Healthy male KM mice (6–8 weeks old) were purchased from SPF (Beijing) Biotechnology Co., Ltd. (Beijing, China). Mice were housed under standard laboratory conditions (temperature: 22 ± 2 °C; 12 h light/dark cycle) with free access to a standard rodent diet (Jiangsu Synergy Biologicals, Nanjing, China) and water. After hair removal, the mice were randomly assigned to different treatment groups. All topical medications were applied by researchers who did not know the group assignments. All animal care and experimental procedures were conducted in strict accordance with the guidelines established by the Institutional Animal Care and Use Committee of Yunnan Agricultural University, and the study protocol was also approved (Approval No. APYNAU 202510002).

#### 4.4.2. SD Model and Experimental Design

An SD model was established in male KM mice. The hair on the dorsal skin was removed using a depilatory cream 24 h before the experiment. Except for the normal group, all groups received UVB irradiation (430 mJ cm^−2^) on the depilated dorsal skin for 72 h. The 2% Carbomer gel used as the vehicle was prepared as follows: 800 mg of Carbomer 940 was added to 40 mL of ultrapure water, and the pH was adjusted with triethanolamine until the gel became transparent. This gel was used as the blank vehicle. For drug-containing gels, the corresponding amounts of PEE, EAE, NBAE, MTX, or LUT were incorporated into the blank gel to achieve the desired doses. The first animal experiment included seven treatment groups: Normal group (no UVB irradiation), Model group (UVB irradiation only), Vehicle Control group (UVB + 2% Carbomer gel), Positive Control group (UVB + 30 mg/mL MTX gel), PEE group (UVB + 60 mg/mL PEE gel), EAE group (UVB + 60 mg/mL EAE gel), and NBAE group (UVB + 60 mg/mL NBAE gel). The second animal experiment also included seven treatment groups: Normal group (no UVB), Model group (UVB irradiation), Vehicle Control group (UVB + 2% Carbomer gel), Positive Control group (UVB + 30 mg/mL MTX gel), Luteolin High-dose group (UVB + 30 mg/mL LUT-H gel), Luteolin Low-dose group (UVB + 15 mg/mL LUT-L gel), and EAE group (UVB + 60 mg/mL EAE gel). All topical treatments were applied once daily for 12 consecutive days. Dorsal skin changes were photographed daily at a fixed time (9:00–10:00 AM), and environmental conditions were kept consistent throughout the experiment.

#### 4.4.3. Histological Analysis

Dorsal skin tissues were embedded in OCT compound and sectioned (8 µm thickness) using a cryostat microtome (CRYOSTAR NX50, Thermo Fisher Scientific, Waltham, MA, USA). Sections were fixed with 4% paraformaldehyde. Haematoxylin and eosin (H&E) staining was performed to evaluate epidermal thickening and inflammatory cell infiltration, and Masson trichrome staining was used to assess collagen fibre deposition in the dermis. Staining procedures strictly followed the manufacturers’ instructions for the respective kits (H&E Staining Kit, Wuhan Servicebio Technology Co., Ltd., Wuhan, China, G1076; Masson’s Trichrome Stain Kit, Wuhan Servicebio Technology Co., Ltd., Wuhan, China, G1006). Stained sections were observed and imaged using a Nikon Eclipse E100 upright optical microscope (Nikon Corporation, Tokyo, Japan) equipped with a Nikon DS-U3 imaging system (Nikon Corporation, Tokyo, Japan).

#### 4.4.4. Enzyme-Linked Immunosorbent Assay (ELISA)

Levels of IL-1β, TNF-α, and MDA in skin tissues were quantified using specific ELISA kits according to the manufacturers’ protocols.

### 4.5. Network Pharmacology Analysis

#### 4.5.1. Screening of Active Compounds and Their Targets

Components identified via UPLC-MS/MS untargeted metabolomics analysis were imported into the Traditional Chinese Medicine Systems Pharmacology (TCMSP) database. OB ≥ 30% and DL ≥ 0.18 were set as dual screening criteria to ensure drug-like potential [40]. The filtered components were then submitted to multiple databases to retrieve potential target genes, including BATMAN-TCM, CTD, HERB, SwissTargetPrediction, and ChEMBL [41,42,43,44,45]. The retrieved targets were integrated and duplicates were removed to establish the active compound–target set.

#### 4.5.2. Collection of Potential Solar Dermatitis Targets

Disease-related targets for “solar dermatitis” were retrieved from the GeneCards, OMIM and DrugBank databases [46,47,48]. After integration and deduplication, the disease target set was established.

#### 4.5.3. Construction of “Drug–Compound–Target–Disease” Network

Cytoscape software (version 3.9.1) was used to construct and visualise the “drug–compound–target–disease” interaction network. Network analysis based on degree centrality was performed to identify core components [49].

#### 4.5.4. Construction of Protein–Protein Interaction (PPI) Network

The STRING database (version 11.5) was used to construct the PPI network for the potential therapeutic targets, with the species limited to Homo sapiens and the interaction confidence threshold set to 0.4. Isolated nodes were excluded from the network [50]. Cytoscape software (version 3.9.1) with its Network Analyzer plugin was employed for topological analysis. The top 14 hub nodes (proteins) with the highest degree values were selected as core targets for subsequent analysis and experimental verification.

#### 4.5.5. Microarray Data Analysis

The GSE54413 dataset was downloaded from the Gene Expression Omnibus (GEO) database [51]. Differentially expressed genes (DEGs) between SD lesions and controls were analysed using the edgeR package (version 3.40.0) in R software (version 4.2.0). Genes with an adjusted *p*-value (FDR) < 0.05 and |log_2_ fold change (log_2_ FC)| ≥ 1 were considered significantly differentially expressed.

#### 4.5.6. Enrichment Analysis

Gene Ontology (GO) functional enrichment analysis and Kyoto Encyclopedia of Genes and Genomes (KEGG) pathway enrichment analysis were performed using R software (version 4.2.0) with the clusterProfiler (version 4.10.0) and org.Hs.eg.db (version 3.17.0) packages to elucidate the biological functions and pathways associated with the potential targets [52]. A *p*-value < 0.05 was considered statistically significant.

### 4.6. Molecular Docking

Molecular docking was performed to investigate the interactions between the major active compounds from EAE and the core target protein. The 3D structures of the ligands (e.g., luteolin) were obtained from the PubChem database. The crystal structure of JUN protein (PDB ID: 3PZE) was downloaded from the RCSB Protein Data Bank. OpenBabel software (version 3.1.1) was used for format conversion. Protein preparation (including hydrogen addition, water removal, and charge assignment) was conducted using AutoDockTools (version 1.5.7). The molecular docking simulations were performed using AutoDock 4.2.6. The grid box was defined using autogrid4, and flexible ligand docking was performed using autodock4. The binding free energy (ΔG, kcal mol^−1^) was calculated to evaluate interaction strength. The optimal docking poses were visualised using PyMOL (version 2.5.0) to highlight key interactions such as hydrogen bonds and hydrophobic contacts.

### 4.7. Molecular Dynamics Simulation

To further validate the binding stability of the optimal ligand–target complex identified by docking, molecular dynamics (MD) simulations were conducted [53]. The complex with a high docking score (>−7.0 kcal mol^−1^) and a stable interaction network (containing ≥3 hydrogen bonds and hydrophobic interactions) was selected. Simulations were performed using GROMACS 2022.3. The GAFF2 force field parameters were assigned to the ligand using AmberTools22, and geometry optimisation and RESP charge calculation were performed at the B3LYP/6-31G* level using Gaussian 16 W. The Amber99sb-ildn force field and TIP3P water model were used for the system. The system was neutralised by adding Na^+^ ions. After energy minimisation (steepest descent, convergence threshold 1000 kJ mol^−1^ nm^−1^), NVT (100 ps, 300 K, V-rescale coupling) and NPT (100 ps, 1 bar, Parrinello–Rahman coupling) equilibration were performed. Finally, a 100 ns production MD simulation was run with a 2 fs time step, saving coordinates every 10 ps. Trajectory analyses included: (1) structural stability assessment via root-mean-square deviation (RMSD) of the protein backbone (converged at <0.3 nm); (2) residual flexibility analysis via root-mean-square fluctuation (RMSF); (3) binding free energy calculation using the MM/PBSA method (sampling every 20 ps); and (4) dynamic interaction network analysis using VMD software. Free energy landscape analysis was also performed to identify dominant conformational clusters.

### 4.8. Cell Experiments

#### 4.8.1. Cell Culture

The human immortalised keratinocyte cell line (HaCaT) was obtained from the Cell Bank of the Key Laboratory of Pu-erh Tea, Ministry of Education, Yunnan Agricultural University. HaCaT cells were cultured in Dulbecco’s Modified Eagle Medium (DMEM) supplemented with 10% foetal bovine serum and 1% penicillin/streptomycin. Cells were maintained at 37 °C in a humidified atmosphere containing 5% CO_2_. Cells were passaged using 0.25% trypsin upon reaching approximately 80% confluence.

#### 4.8.2. Cell Proliferation Assay

Cell proliferation was assessed using the MTT assay. HaCaT cells in the logarithmic growth phase were seeded into 96-well plates at a density of 2 × 10^4^ cells per well and incubated overnight. Cells were then treated with various concentrations of luteolin or EAE for 24 h. Subsequently, 20 µL of MTT solution (5 mg mL^−1^) was added to each well and incubated for 4 h. The supernatant was carefully removed, and 200 µL of DMSO was added to dissolve the formazan crystals. Absorbance was measured at 490 nm using a microplate reader.

#### 4.8.3. Establishment of Cell Model and Experimental Design

HaCaT cells were seeded into 60 mm cell culture dishes at 70% confluence. When cells reached about 70% confluence, the medium was replaced with serum-free DMEM for 18 h of starvation. Then, cells were treated with different agents for 4 h. For the UVB irradiation groups, after incubation with the agents, part of the medium was discarded, and cells were exposed to UVB lamps for 6 h. After irradiation, cells were placed on ice for 5 min and then lysed for protein extraction.

The experiment was divided into two conditions: without UVB irradiation and with UVB irradiation.

Without UVB irradiation: Cells were divided into 7 groups: Blank Control (serum-free medium only), JUN Activator group (Anisomycin, 20 µM), JUN Inhibitor group (SP600125, 8 µM), JUN Activator + Inhibitor group, JUN Activator + MTX group (Anisomycin + MTX, 20 µM), JUN Activator + Luteolin group (Anisomycin + Luteolin, 12.5 µM), and JUN Activator + EAE group (Anisomycin + EAE, 150 µg mL^−1^).

With UVB irradiation: Cells were divided into 6 groups: Blank Control (serum-free medium only), UVB group (UVB irradiation only), UVB + JUN Inhibitor group (UVB + SP600125), UVB + MTX group (UVB + MTX), UVB + Luteolin group (UVB + Luteolin), and UVB + EAE group (UVB + EAE).

### 4.9. Western Blotting Analysis

Total protein was extracted from dorsal skin tissues or HaCaT cells using ice-cold RIPA lysis buffer. Tissue homogenates or cell lysates were centrifuged at 12,000 *g* for 12 min at 4 °C, and the supernatants were collected. Protein concentration was determined using the BCA protein assay kit. Equal amounts of protein were separated by 8–12% SDS-PAGE and transferred onto PVDF membranes. The membranes were blocked and then incubated overnight at 4 °C with primary antibodies against JUN, p-JUN, IL-1β, TNF-α, and β-tubulin (as a loading control). After washing, membranes were incubated with HRP-conjugated secondary antibodies for 1.5 h at room temperature. Protein bands were visualised using enhanced chemiluminescence (ECL) substrate and detected with a ChemiDoc MP Imaging System (Sevier Biologics, Wuhan, China). Band intensities were quantified using ImageJ software (version 7.0).

### 4.10. Statistical Analysis

All data are presented as mean ± standard deviation (SD). Statistical analysis was performed using Prism 10 (GraphPad Software, San Diego, CA, USA). Differences between groups were analysed using an unpaired *t*-test or one-way analysis of variance (ANOVA), followed by Dunnett’s multiple comparisons test when ANOVA indicated significant differences. A *p*-value < 0.05 was considered statistically significant.

## 5. Conclusions

This study, through the integration of metabolomics, network pharmacology, molecular docking, molecular dynamics simulation, and in vitro and in vivo experimental validation, elucidated that EAE likely treats UVB-induced SD through a multi-component, multi-target approach, with LUT being a key active substance in EAE. In vivo experiments demonstrated that both EAE and LUT significantly improved erythema, oedema, epidermal thickening, and collagen damage in SD mice, reduced the levels of pro-inflammatory factors TNF-α and IL-1β and the oxidative stress marker MDA, and promoted skin barrier function recovery. Through network pharmacology screening combined with GEO dataset cross-validation, molecular docking, and molecular dynamics simulations, JUN was identified as the core target for EAE in treating SD. In vitro and in vivo experimental results consistently showed that both LUT and EAE could inhibit the abnormal activation of the JUN pathway and downregulate the overexpression of TNF-α and IL-1β. Simultaneously, LUT reversed UVB-induced skin metabolic disturbances and promoted tissue repair by regulating pathways such as glutathione metabolism and the pentose phosphate pathway. In summary, EAE and its core active component LUT likely exert therapeutic effects on SD by inhibiting JUN signalling pathway-mediated inflammatory and oxidative stress responses and improving the skin metabolic microenvironment. This study provides a solid theoretical and experimental basis for developing novel, highly effective, and low-toxicity natural anti-photodamage agents.

## Figures and Tables

**Table 1:** Six candidate bioactive compounds selected from the unique components of the EAE of GEE based on drug-likeness criteria.

Compound	Formula	tR (min)	HMDB ID	Molecular Weight	OB (%)	DL
Aloe-emodin	C_15_H_10_O_5_	5.522	HMDB0030829	270.05282	83.38	0.24
Eriodictyol	C_15_H_12_O_6_	3.9	HMDB0005810	288.06339	71.79	0.24
Luteolin	C_15_H_10_O_6_	2.721	HMDB0005800	286.04774	36.16	0.25
Quercetin	C_15_H_10_O_7_	2.07	HMDB0005794	302.04265	46.43	0.28
Skrofulein	C_17_H_14_O_6_	4.49	HMDB0250276	314.07904	30.35	0.30
Wogonin	C_16_H_12_O_5_	4.372	--	284.06847	30.68	0.23

--, not available.

## Data Availability

The original contributions presented in the study are included in the article/Supplementary Materials; further inquiries can be directed to the corresponding authors.

## Supplementary Materials

The following supporting information can be downloaded at: https://www.mdpi.com/article/10.3390/ph19071042/s1. Supplementary Figure S1: Dorsal skin changes in mice from day 0 to 12. Supplementary Figure S2: PCA of PEE group, EAE group, and NBAE group. Supplementary Figure S3: Total ion chromatograms (TIC) in negative electrospray ionization mode (ESI−) of untargeted metabolomics analysis for the PEE, EAE and NBAE fractions of GE. Supplementary Figure S4: Total ion chromatograms (TIC) in positive electrospray ionization mode (ESI+) of untargeted metabolomics analysis for the petroleum PEE, EAE and NBAE fractions of GE. Supplementary Figure S5: Analysis of the active constituents in GE fractions of different polarities. (A) Percentage and quantity of non-volatile substances in PEE. (B) Percentage and quantity of non-volatile substances in EAE. (C) Percentage and quantity of non-volatile substances in NBAE. Supplementary Figure S6: Volcano plot of the GSE54413 dataset. Supplementary Figure S7: Extracted ion chromatograms (XIC) and mass spectrometry identification of LUT in GE polarity fractions. (A–C) XIC of LUT in PEE, EAE, and NBAE, respectively. (D) Primary mass spectrum (MS1). Supplementary Figure S8: MTT results of the EAE group. Supplementary Figure S9: MTT results of the LUT group. Supplementary Figure S10: Changes in the dorsal skin of mice from day 0 to day 12.
