# Supplementary material for: Luteolin from Gastrodia elata Ameliorates Solar Dermatitis via Inhibition of the MAPK/JUN Signalling Pathway
Source: Pharmaceuticals (Basel). 2026 Jul 3;19(7):1042. doi: 10.3390/ph19071042 (PMC13416056; doi:10.3390/ph19071042)
Supplement: Supplementary file 1 [file pharmaceuticals-19-01042-s001.zip › pharmaceuticals-4338199-supplementary.pdf]

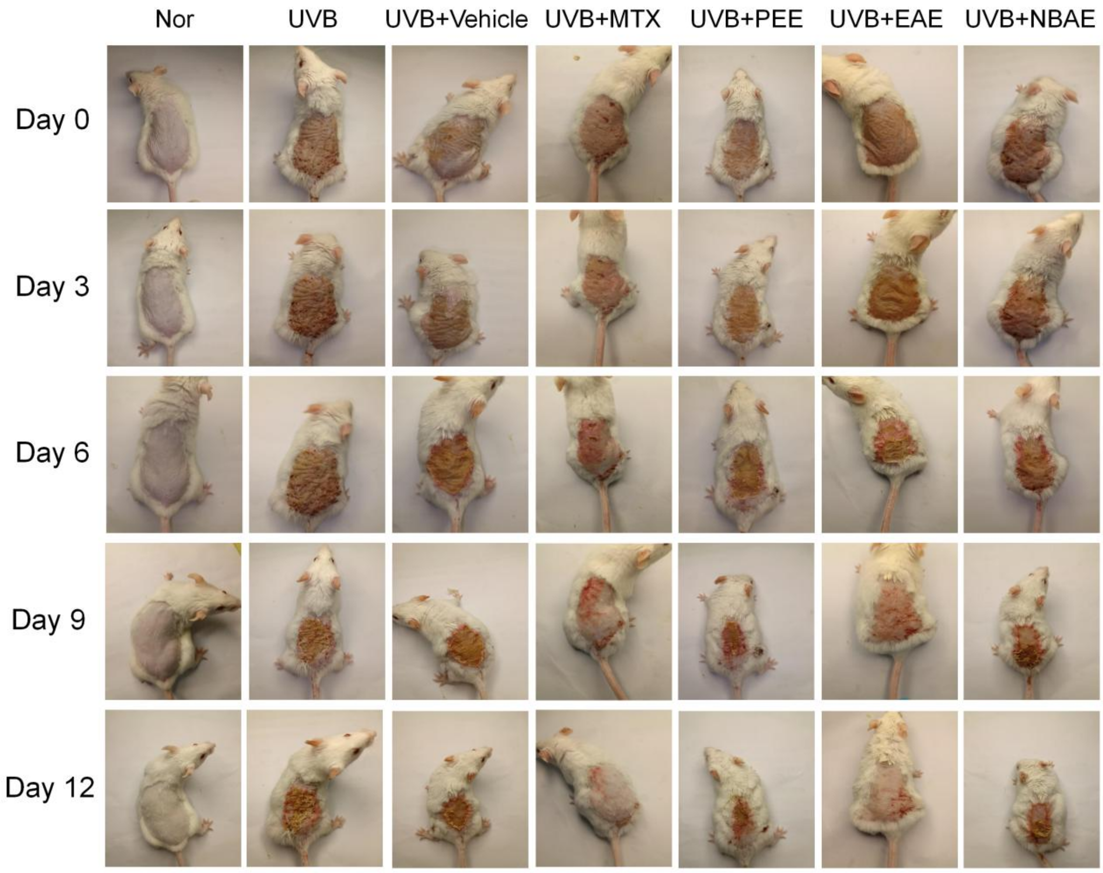


**Supplementary Figure S1** Dorsal skin changes in mice from day 0 to 12.

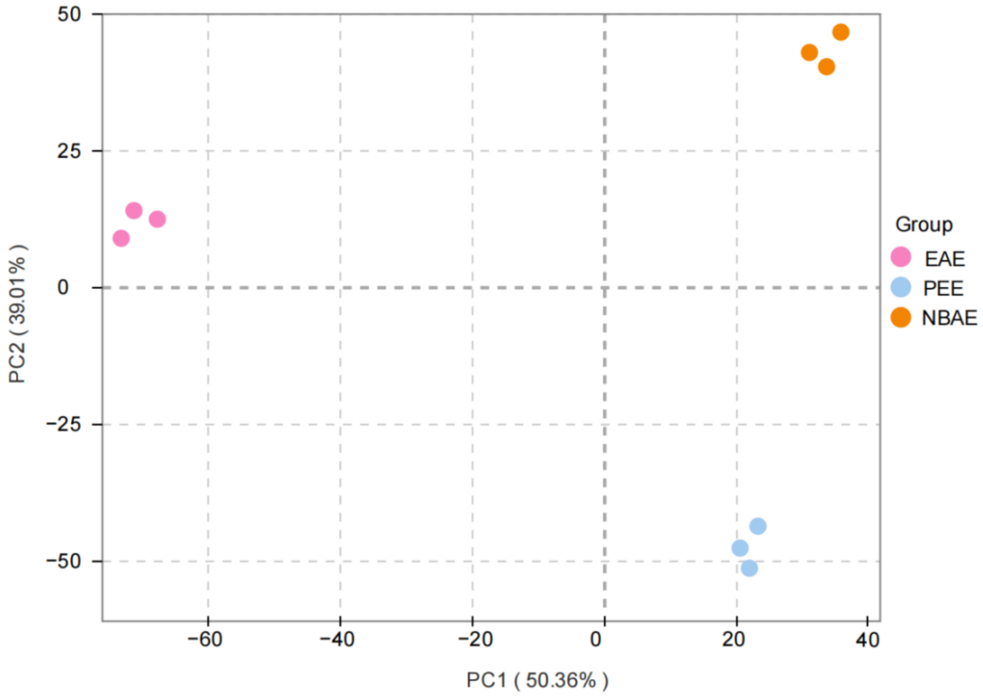


**Supplementary Figure S2** PCA of PEE group, EAE group, and NBAE group

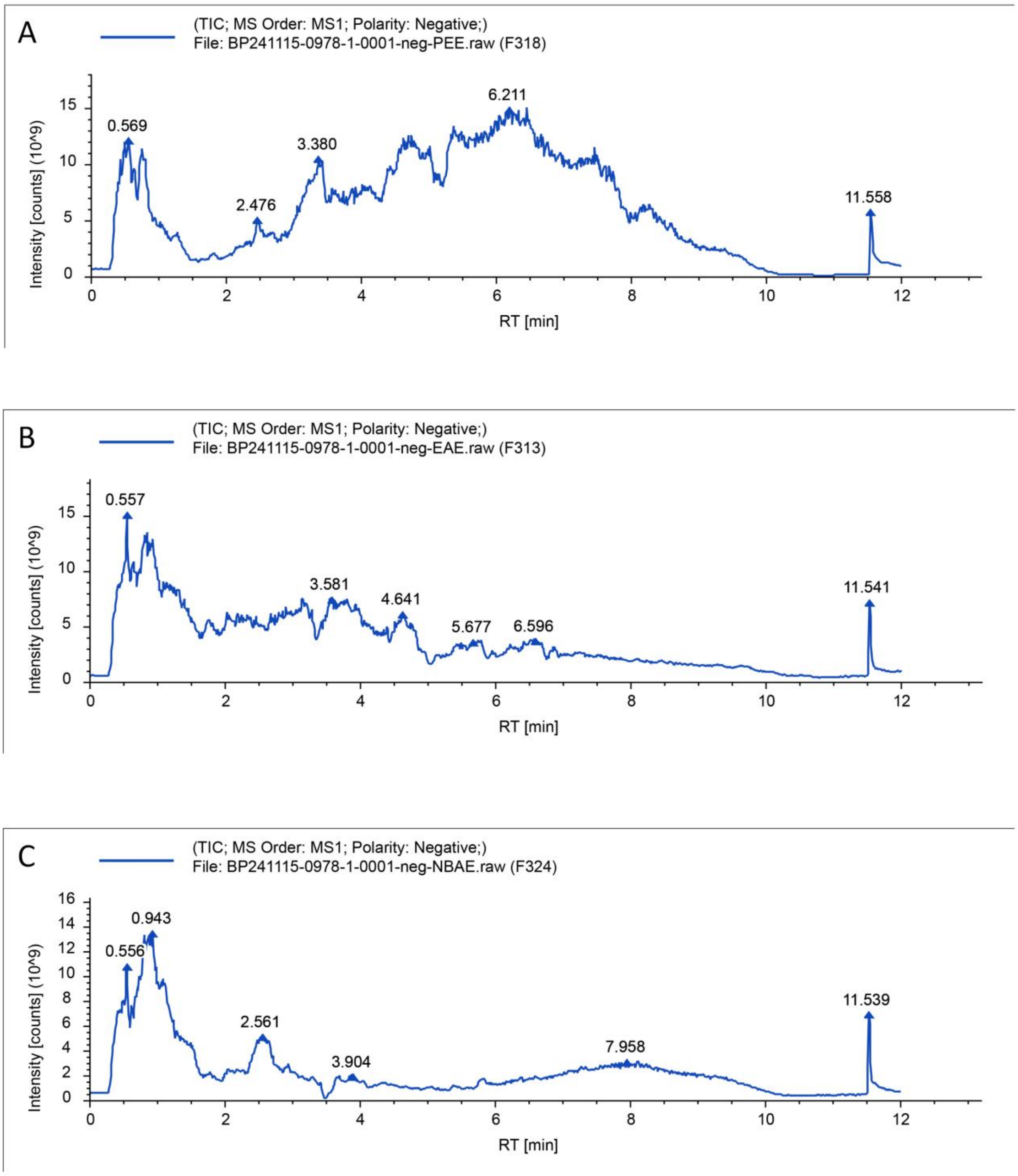


**Supplementary Figure S3** Total ion chromatograms (TIC) in negative electrospray ionization mode (ESI⁻) of untargeted metabolomics analysis for the PEE, EAE and NBAE fractions of GE.

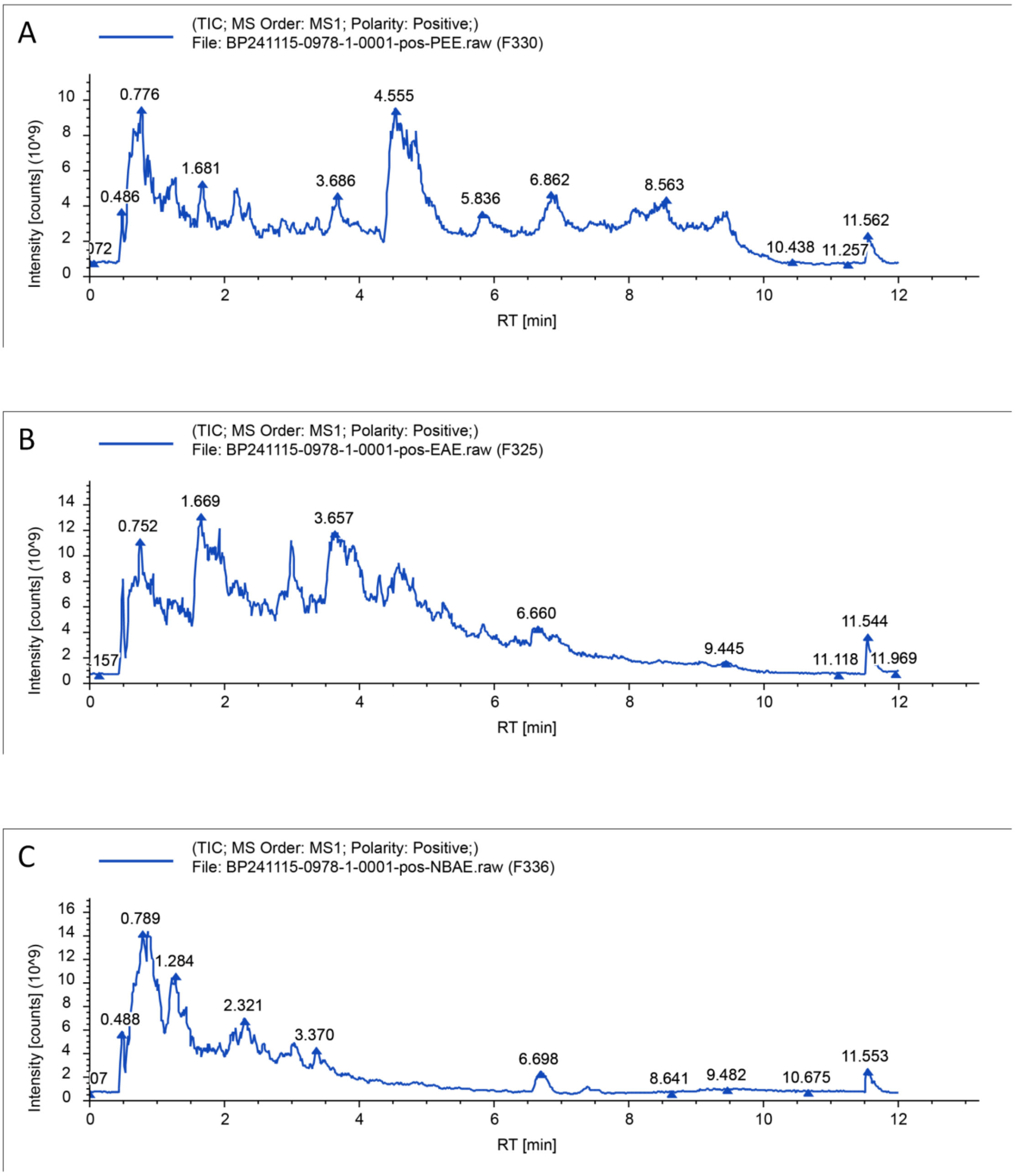


**Supplementary Figure S4** Total ion chromatograms (TIC) in positive electrospray ionization mode ($ESI^+$) of untargeted metabolomics analysis for the petroleum PEE, EAE and NBAE fractions of GE.

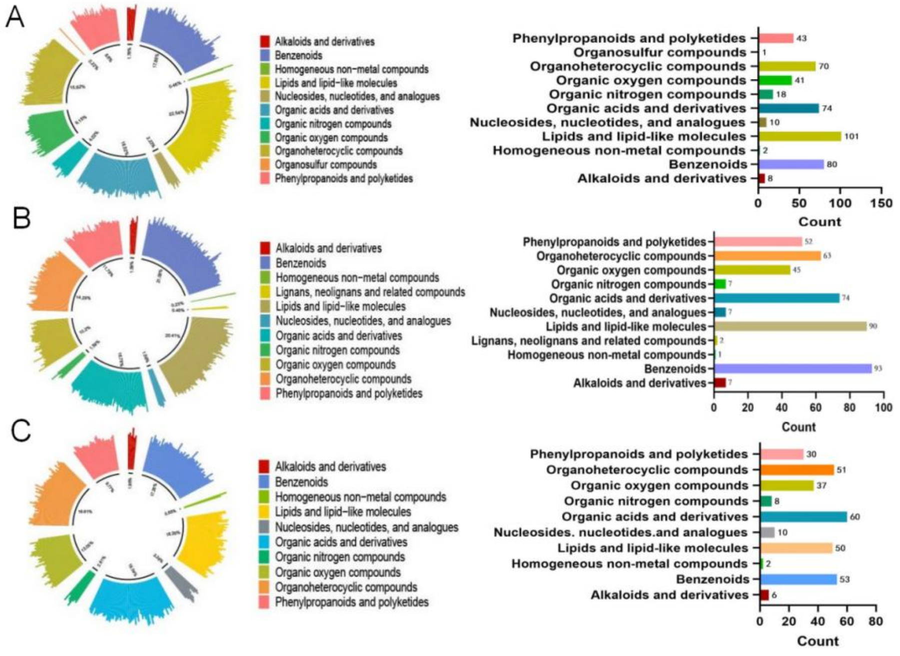


**Supplementary Figure S5** Analysis of the active constituents in GE fractions of different polarities. (A) Percentage and quantity of non-volatile substances in PEE. (B) Percentage and quantity of non-volatile substances in EAE. (C) Percentage and quantity of non-volatile substances in NBAE.

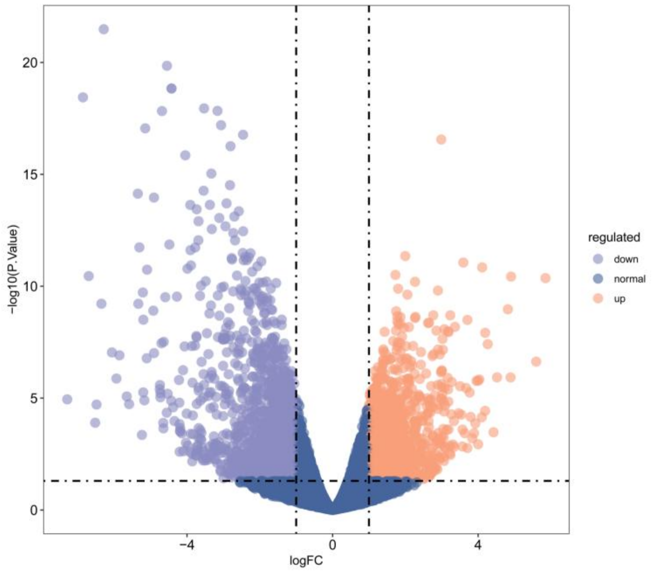


**Supplementary Figure S6** Volcano plot of the GSE54413 dataset.

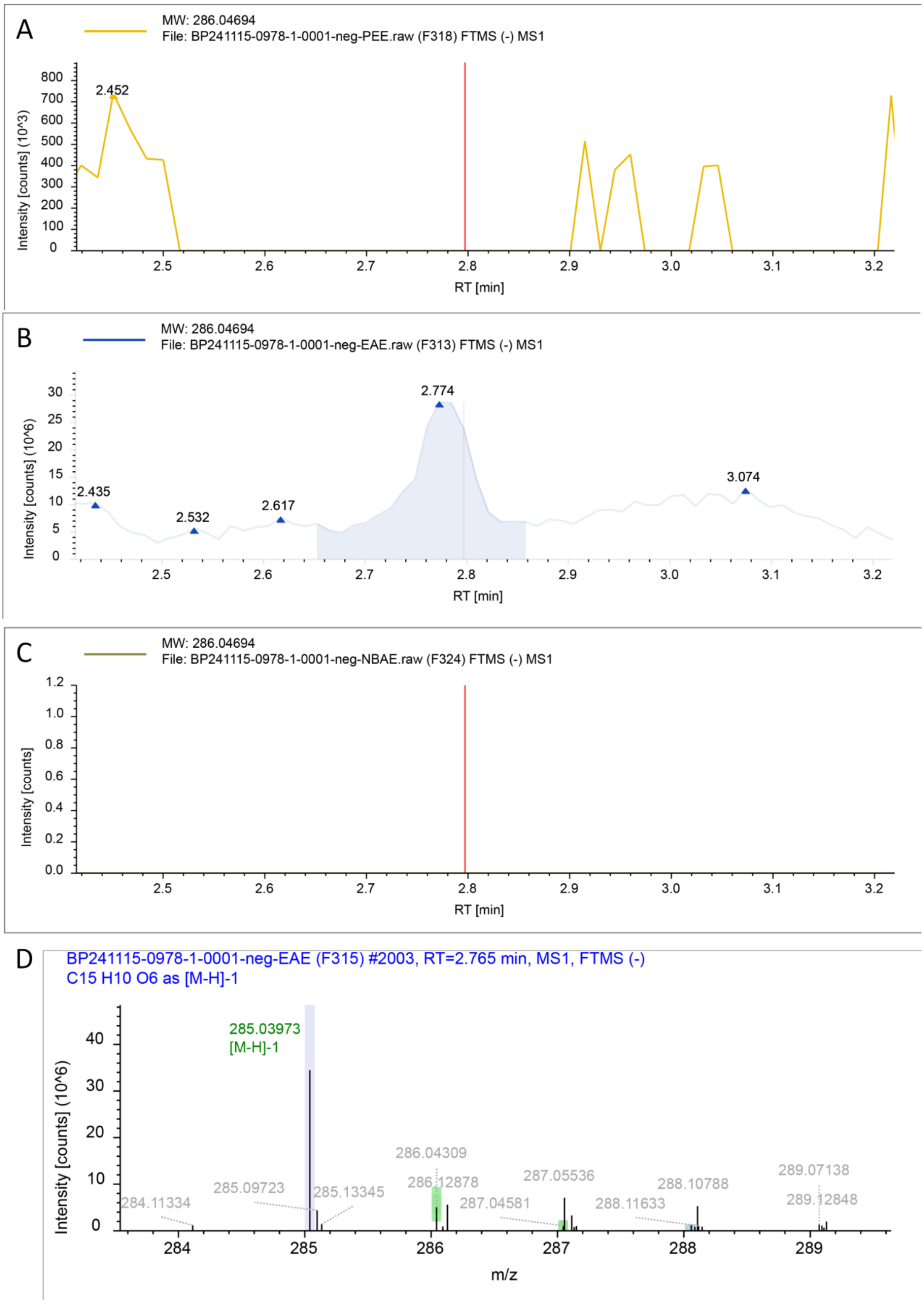


**Supplementary Figure S7** Extracted ion chromatograms (XIC) and mass spectrometry identification of LUT in GE polarity fractions. (A-C) XIC of LUT in PEE, EAE, and NBAE, respectively. (D) Primary mass spectrum (MS¹) .

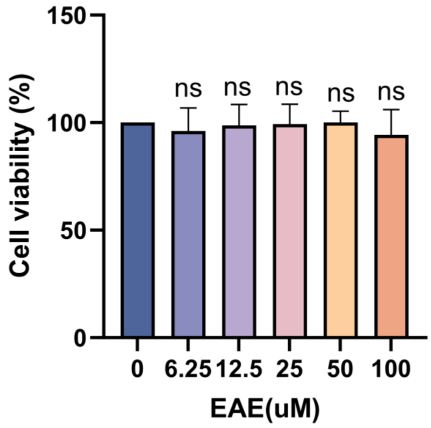


**Supplementary Figure S8** MTT results of the EAE group.

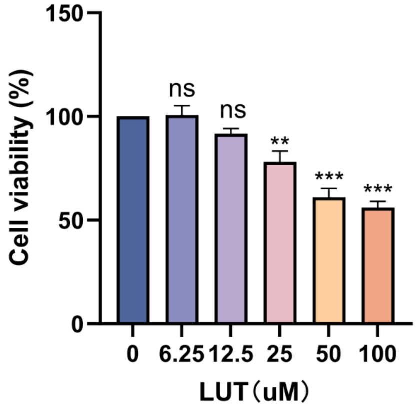


**Supplementary Figure S9** MTT results of the LUT group.

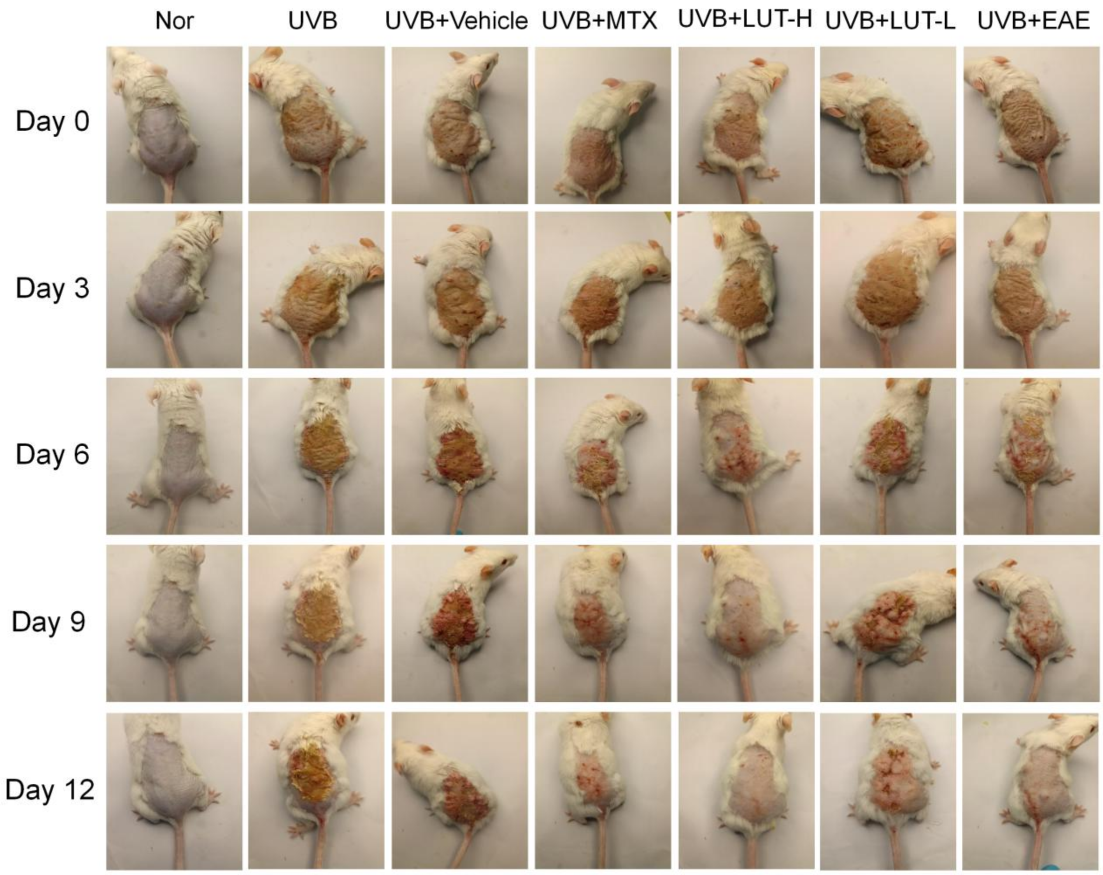


**Supplementary Figure S10** Changes in the dorsal skin of mice from day 0 to day 12.
